# Harnessing UAVs and deep learning for accurate grass weed detection in wheat fields: a study on biomass and yield implications

**DOI:** 10.1186/s13007-024-01272-6

**Published:** 2024-09-19

**Authors:** Tao Liu, Yuanyuan Zhao, Hui Wang, Wei Wu, Tianle Yang, Weijun Zhang, Shaolong Zhu, Chengming Sun, Zhaosheng Yao

**Affiliations:** 1 Cultivation and Construction Site of National Key Laboratory for Crop Genetics and Physiology in Jiangsu Province, Yangzhou University, Yangzhou, 225009 PR China; 2 Lixiahe Institute of Agricultural Sciences, Jiangsu, Yangzhou, 225007 China; 3 Key Laboratory of Agro-information Services Technology, Ministry of Agriculture, Beijing, 100081 China

**Keywords:** Grass weeds, Wheat field, Unmanned aerial vehicles (UAVs), Deep learning algorithm, Hyperspectral imaging

## Abstract

Weeds are undesired plants competing with crops for light, nutrients, and water, negatively impacting crop growth. Identifying weeds in wheat fields accurately is important for precise pesticide spraying and targeted weed control. Grass weeds in their early growth stages look very similar to wheat seedlings, making them difficult to identify. In this study, we focused on wheat fields with varying levels of grass weed infestation and used unmanned aerial vehicles (UAVs) to obtain images. By utilizing deep learning algorithms and spectral analysis technology, the weeds were identified and extracted accurately from wheat fields. Our results showed that the precision of weed detection in scattered wheat fields was 91.27% and 87.51% in drilled wheat fields. Compared to areas without weeds, the increase in weed density led to a decrease in wheat biomass, with the maximum biomass decreasing by 71%. The effect of weed density on yield was similar, with the maximum yield decreasing by 4320 kg·ha^− 1^, a drop of 60%. In this study, a method for monitoring weed occurrence in wheat fields was established, and the effects of weeds on wheat growth in different growth periods and weed densities were studied by accurately extracting weeds from wheat fields. The results can provide a reference for weed control and hazard assessment research.

## Introduction

Weeds are unwanted plants that compete with crops in terms of light, nutrition, water, and growth space. Weeds can lead to the deterioration of the field environment, and inhibit the growth and development of wheat, resulting in slow growth, stunted growth, and reduced yield and quality [1, 2]. A large infestation of weeds can cause a 60% reduction in wheat yield. The degree of yield reduction mainly depends on the density of weeds and the length of time of weed interference [3]. Monitoring weeds is essential for targeted control and precise spraying, which can help reduce pesticide consumption and non-point source pollution while improving the accuracy and effectiveness of field management. Therefore, it is important to investigate effective weed identification methods in wheat fields to achieve precise pesticide spraying and targeted weed control in future experiments. Grass weeds are among the most challenging weeds to control in wheat fields and represent the primary weed species during the wheat’s overwintering and jointing stages.

The current research methods for weed monitoring can be divided into artificial recognition, machine vision, various image acquisition technologies, and different image extraction methods. Ashraf and Khan [4] used two methods to separate weeds and crops: one classified weeds based on texture features, and the other classified weed images based on plant shape and anatomical structure. Sabzi and Sajad [5] used machine vision technology to segment potato plants and weeds; their method achieved correct detection, segmentation, and classification of potato plants and weeds at a speed of 0.15 m·s^-1^. Raja et al. [6] used machine vision technology and crop signaling techniques to distinguish crops and weeds. Raveendra et al. [7] demonstrated the capability of LabVIEW in performing machine vision to reduce human intervention in agricultural production. Their method can process the digital images acquired by the camera and match the captured weed leaves with a hypothetical database for health confirmation. Jiang et al. [8] proposed a weed detection method based on Mask R-CNN (a framework for image segmentation tasks), which combines target detection and semantic segmentation to classify weeds and crops while detecting weeds. In the above studies, RGB images were mostly used for weed identification. The use of hyperspectral images in weed recognition tasks can provide more information about the targets than RGB images. It is also very beneficial for improving recognition accuracy. Li et al. [9] constructed a classification of perennial weeds based on hyperspectral data. Pan et al. [10] used a variety of preprocessing and characteristic wavelength extraction methods to process the canopy hyperspectral data of plants and weeds, analyzed the spectra of different bands to establish the average reflectance spectrum curve of plants and weeds, and finally distinguished weeds and plants through principal component analysis.

The UAV remote sensing systems have clear advantages, including being fast, non-destructive, low cost, and high throughput. In recent years, UAV remote sensing technology has been gradually applied to monitoring crop growth in agriculture [11]. The UAV remote sensing system comprises UAVs and micro-miniature multispectral and hyperspectral sensors, which can obtain high-resolution images [12]. Zhao et al. [13] used the UAV remote sensing platform to obtain the characteristics of plants and weeds at the canopy scale, divided the vegetation and non-vegetation areas in the image by the maximum between-class variance method, and segmented the weed areas. Using hyperspectral and deep learning techniques can significantly improve the classification of farmland vegetation in UAV images [14]. The method also provides a reference for the identification of weeds.

In wheat cultivation, the predominant weed species can be categorized into members of the Poaceae family (commonly referred to as grass weeds) and broadleaf weeds. The foliar morphology of broadleaf weeds markedly differs from that of wheat seedlings, facilitating their identification. Conversely, grass weeds display considerable morphological similarities to wheat seedlings, complicating their differentiation. Although previous research has exten- sively addressed the identification of broadleaf weeds within wheat fields, studies focusing on the identification of Poaceae weeds remain limited. There are also few published studies on the use of UAVs for monitoring weeds in wheat fields. This is primarily due to the minimal differences in color and spectral characteristics between grass weeds and wheat, making it challenging to distinguish between them. In this study, we focused on wheat fields with varying levels of weed infestation, acquired UAV images, and investigated the impact of different growth stages and weed densities on wheat growth. This research aims to provide valuable insights for weed management and risk assessment. The specific objectives of this study are as follows:

1. To develop an identification model for grass weeds in wheat fields based on deep learning algorithms, pinpointing the locations of weeds;
2. Construct a weed biomass estimation model utilizing hyperspectral imagery to assess the prevalence of weed infestations;
3. Evaluate the impact of different models on the accuracy of weed identification;
4. Investigate wheat yield data under various weed infestation scenarios and assess the implications of weed-induced damage.

## Materials and methods

### Data acquisition

**Fig. 1:**
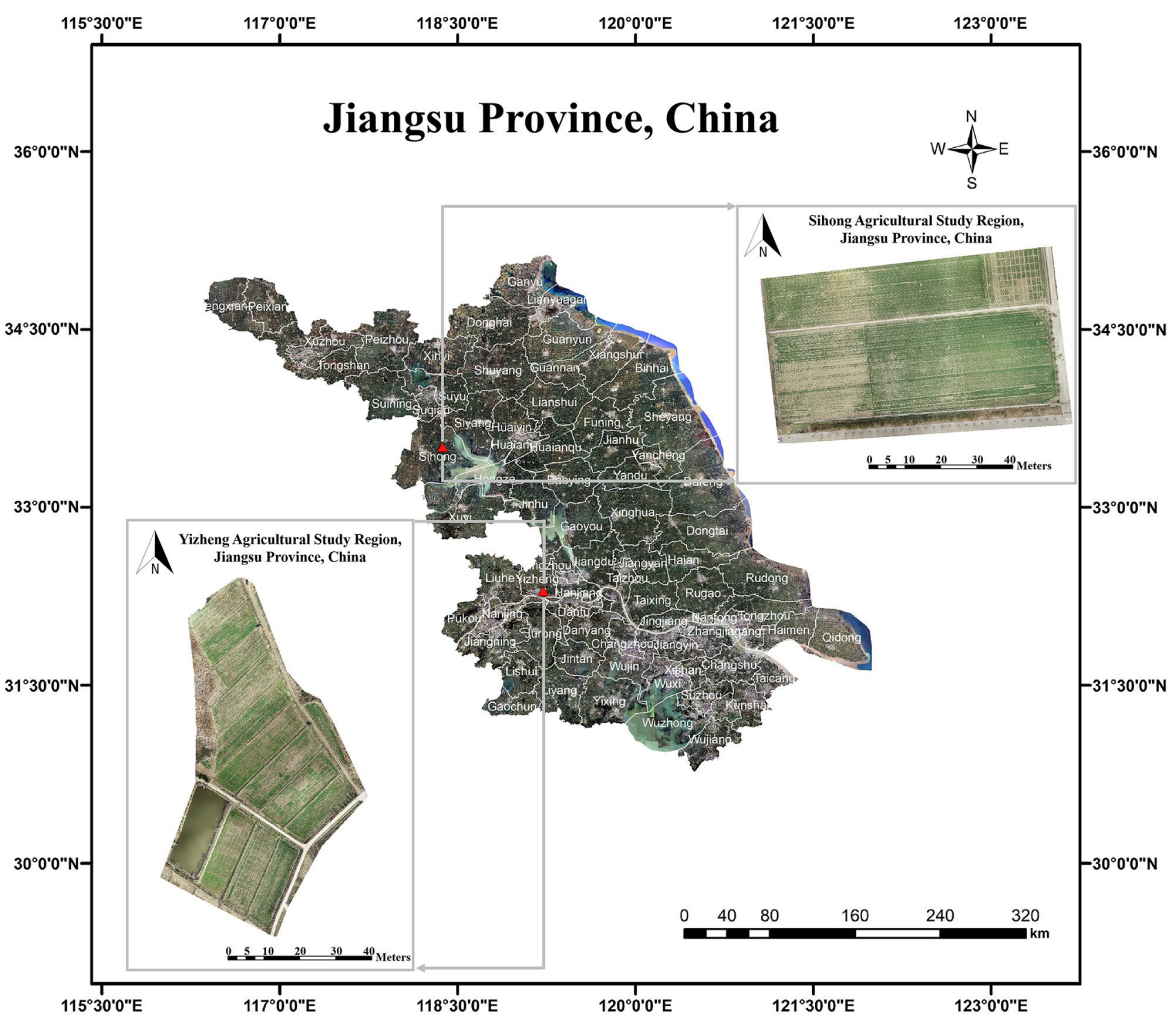
Study region

Our study was conducted in Yizheng and Sihong, Jiangsu Province, China, in 2019–2020 (Fig. 1). Eight test plots were selected as the research subjects, of which six plots had varying degrees of weed infestation, while the remaining two plots were kept weed-free as control. The species of weed are *Alopecurus* pratensis (*Alopecurus aequalis Sobol.*) and *Poa* pratensis (*Poa annua L.*). The images were obtained by UAVs during the wintering, seedling, and jointing periods. The sensor parameters used are shown in Table 1. The images were acquired on sunny days between 10:00 a.m. and 2:00 p.m. In hyperspectral image acquisition, there was a 50% overlap between routes and a 60% overlap between waypoints. The corresponding percentages were 70% and 80% in multispectral image acquisition. The images of the standard gray cloths were obtained during image acquisition.

Field sampling and image acquisition of weeds and wheat were carried out simultaneously. The collected samples were fixed at 105℃ for 30 min and then dried at 80℃ to constant weight. The above-ground biomass of both wheat and weeds were measured, respectively.

**Table 1:** Parameters of the image acquisition equipment

Equipment	Camera technology	Flight altitude
DJI Matrice 600 Pro + GaiaSky-mini Camera	Wavelength range: 400–1000 nm Spectral resolution: 3.5 nm Image resolution: 1920 × 2080 Weight: 1.5 kg	50 m
DJI Mavic 3	Positioning accuracy: 1–1.5 cm CMOS: Mavic 3 Image resolution: 5280 × 3956 Field of view: 84°	15 m

### Data processing

**Fig. 2:**
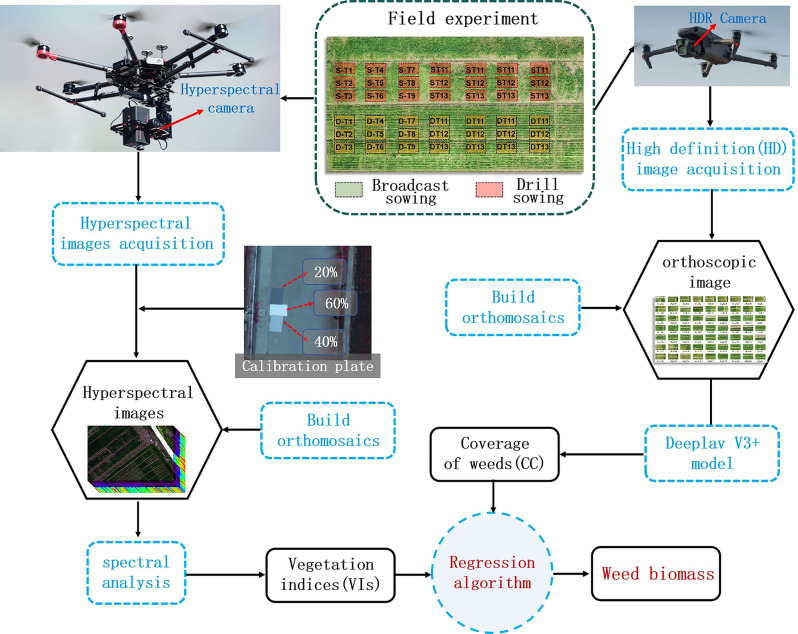
The flow chart of this research

After lens calibration, reflectance calibration, and atmospheric calibration through SpecView software (Jiangsu Dualix Spectral Imaging Technology Co., Ltd. China), hyperspectral images were stitched through HiSpectralStitcher software (Jiangsu Dualix Spectral Imaging Technology Co., Ltd. China). RGB images were stitched through Metashape software (Agisoft LLC, Russia) to generate orthophotos images, and the accuracy of image stitching was further improved through ground control points. Vegetation indexes, i.e., normalized difference vegetation index (NDVI), ratio vegetation index (RVI), and soil-adjusted vegetation index (SAVI), were extracted on the acquired images [15]. The processing flow is shown in Fig. 2. All experiments were conducted on a high-performance computing setup with the following hardware and software specifications: CPU: Intel Core i7-9700 K, 8 cores; GPU: NVIDIA GeForce RTX 3090, 24GB VRAM; Memory (RAM): 64GB DDR4; Storage: 1 TB NVMe SSD.

### Semantic segmentation algorithm

**Fig. 3:**
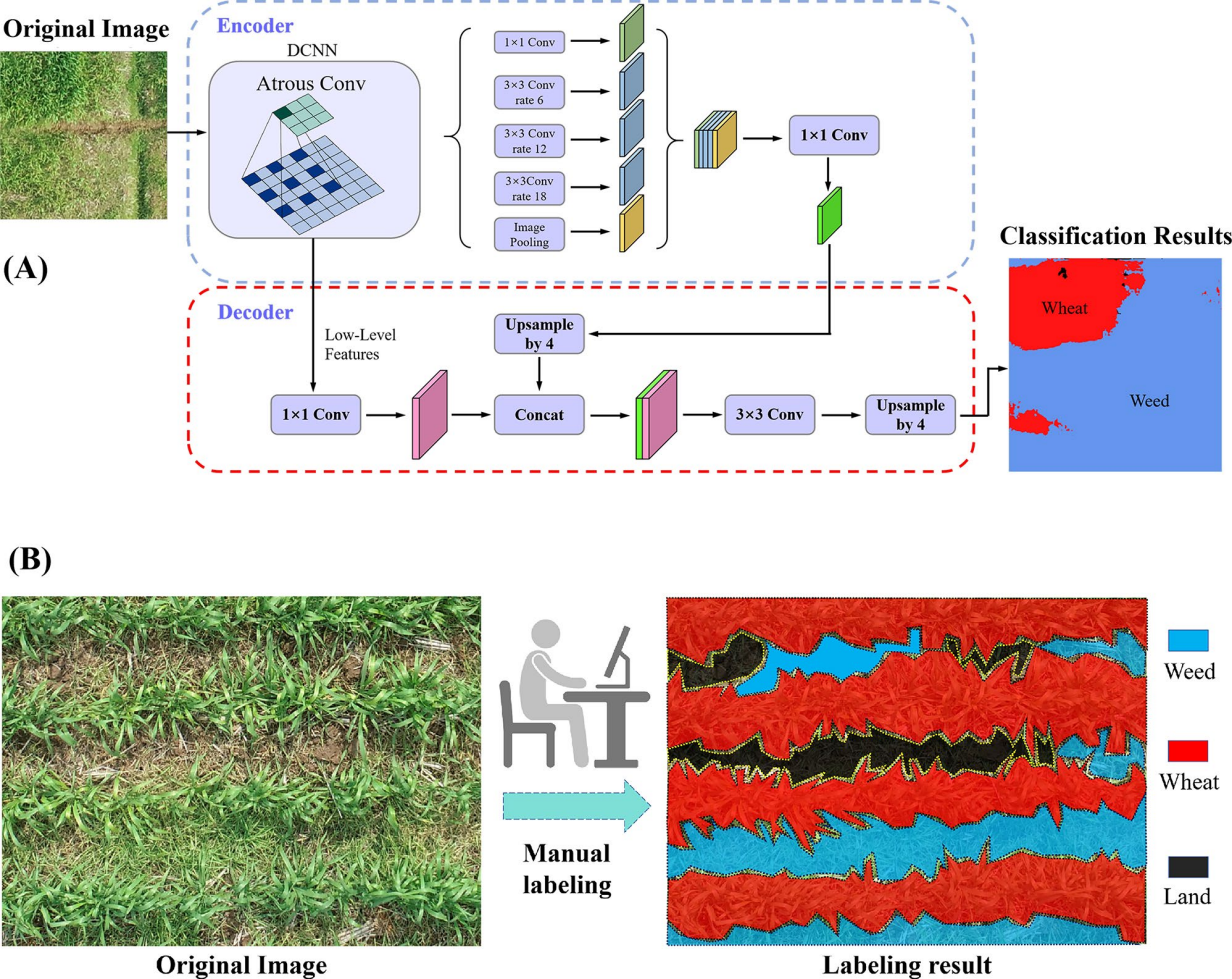
Extracting weed coverage using deep learning. (**A**) The network structure of deep learning; (**B**) The labeling processes and results

In this research, DeepLabV3 + was used to segment weeds and wheat. Based on the DeepLabV3 + framework of deep learning, using the Encoder-Decoder structure has a relatively better effect in boundary extraction applications, which can significantly improve the accuracy of image segmentation [16, 17]. The encoder uses the ResNet-50 residual network as a skeleton network for feature extraction. ResNet-50 introduces the components of residual modules to deepen network depth but does not add additional parameters and calculations to the network. In addition, compared with VGGNet, it increases the training speed and obtains better training effects and model accuracy. First, the first three groups of residual blocks of ResNet-50 are serially concatenated, and then the fourth group of residual blocks is modified to use dilated convolution. Atrous spatial pyramid pooling (ASPP) is introduced, which can capture multi-scale information through different sampling rates and aggregate the prediction results from five parallel branches to obtain a 1 × 1 convolution feature map, which is then input to the decoder for use. The decoder draws on fully convolutional networks (FCNs) characteristics by combining low-level feature and high-level feature prediction. In the detailed information of low-level features passing through, to reduce the number of channels, we first use 48-channel 1 × 1 convolution to convolve the low-level feature map to obtain the prediction map. We then fuse it with the high-level feature map that is upsampled at 4-fold and perform a 3 × 3 convolution operation. The original resolution is restored after upsampling at 4-fold again. Finally, we use the Softmax classifier to classify each pixel to obtain the pixel classification probability map. The principle is shown in Fig. 3A.

A total of 1,000 RGB images of wheat field weeds in the wintering period and jointing period were collected during this study, and 10,000 sub-images were cropped into 318 × 318 pixels, of which 7,000 images were used for the simple semantic segmentation model, and 3,000 images were used for verification. The training images were manually annotated using LabelMe software (MIT, Computer Science and Artificial Intelligence Laboratory, USA), as shown in Fig. 3B, where red labeled areas indicate wheat, blue indicates weeds, and black indicates soil. The model was developed based on Python 3.7. The study employs the deep learning model to perform weed extraction, consequently calculating the weed canopy cover (CC) for weed biomass estimation.

### Accuracy evaluation

One of the important indicators to measure the accuracy of the remote sensing image semantic segmentation algorithm is Mean Intersection over Union (MIoU), which is the average of the ratio of the intersection and the union of the ground truth and predicted segmentation sets, i.e., the average value after the sum of Intersection over Union (IoU) of each type of ground feature. Identifying wheat field weeds is a three-class issue (wheat, weeds, and land). We use F1 values, precision, and recall as the accuracy evaluation indicators. The specific calculation formulas are as follows:

1 $$\:\begin{array}{c}IoU=\frac{TP}{FN+FP+TP}\end{array}$$

2 $$\:\begin{array}{c}MIoU=\frac{1}{k+1}{\sum}_{i=0}^{k}\frac{TP}{FN+FP+TP}\end{array}$$

3 $$\:\begin{array}{c}F1=\frac{2\times{TP}}{N+TP-TN}\end{array}$$

4 $$\:\begin{array}{c}\text{Precision}=\frac{TP}{TP+FP}\end{array}$$

5 $$\:\begin{array}{c}\text{R}\text{e}\text{c}\text{a}\text{l}\text{l}=\frac{TP}{TP+FN}\end{array}$$

where TP is true positive, TN is true negative, FN is false negative, FP is false positive, N is the total number of samples, and k is the segmentation of k-type ground features.

The coefficient of determination (R^2^) values of the models, the root mean square error (RMSE), and the relative error in prediction (REP) are used to test the performance of the model. R^2^ and RMSE are used to describe the stability of the model and the average deviation of the measured value from the true value.

### Modeling and validation

The results of previous showed that regression model such as linear regression, partial least squares regression (PLSR), Lasso regression, support vector regression (SVR) and many other regression methods could be used to estimate these agronomy parameters like biomass, LAI and the content of nitrogen. In this study, the SVR algorithm is employed to construct a weed biomass estimation model. This study compared the effects of common regression models with or without weeds CC calculated by DeepLab V3+. RMSE and R^2^ were used for model evaluation. Of the field data, 50% of 2022 and 2023 data were selected for modeling and 50% for validation.

6 $$\:\begin{array}{c}R\text{MSE}=\sqrt{\frac{1}{\text{\:m}}{\sum}_{i=1}^{m}{\left(p{y}_{i}-t{y}_{i}\right)}^{2}}\end{array}$$

7 $$\:\begin{array}{c}SSE={\sum}_{i=1}^{m}{\left(p{y}_{i}-{\text{t}\text{y}}_{i}\right)}^{2}\:\end{array}$$

8 $$\:\begin{array}{c}SST={\sum}_{i=1}^{m}{\left({\text{t}\text{y}}_{i}-\bar{\text{y}}\right)}^{2}\end{array}$$

9 $$\:\begin{array}{c}{R}^{2}=1-\frac{SSE}{\text{S}\text{S}\text{T}}\end{array}$$

where m is the sample size, py_i_ is the predicted value of model, ty_i_ is the true value, and $\bar{\text{y}}$ is the mean of the observed data from the field.

## Results

### The characteristics of weeds and wheat

**Fig. 4:**
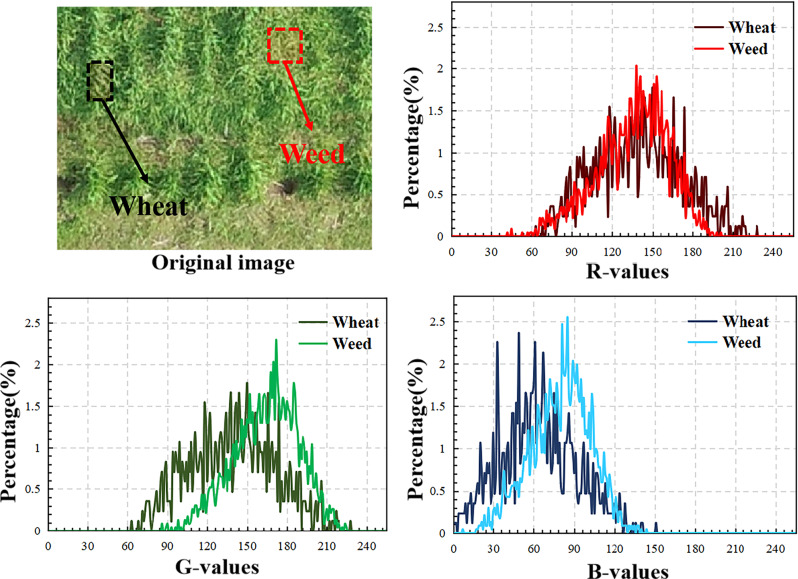
Histogram of wheat and weeds

The fundamental reason for the difficulty in identifying weeds in wheat fields is that the color difference between weeds and wheat is small. As shown in Fig. 4, in the RGB image, the histograms of weed and wheat on the three channels of red (R), green (G), and blue (B) are very similar, so it is very difficult to distinguish weeds from wheat directly using color images.

**Fig. 5:**
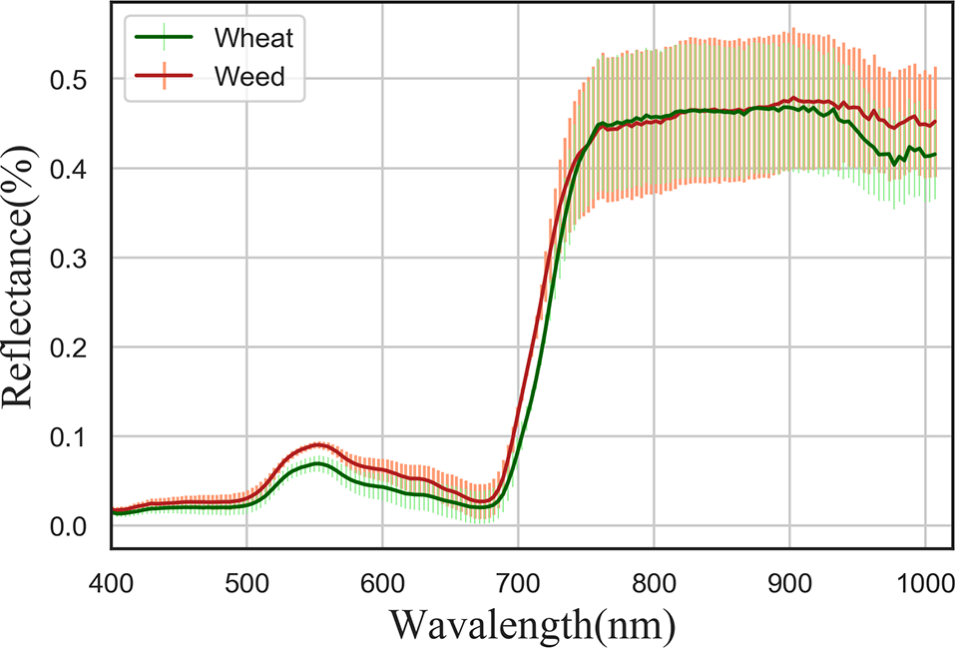
Reflectance curve of wheat and weeds

**Fig. 6:**
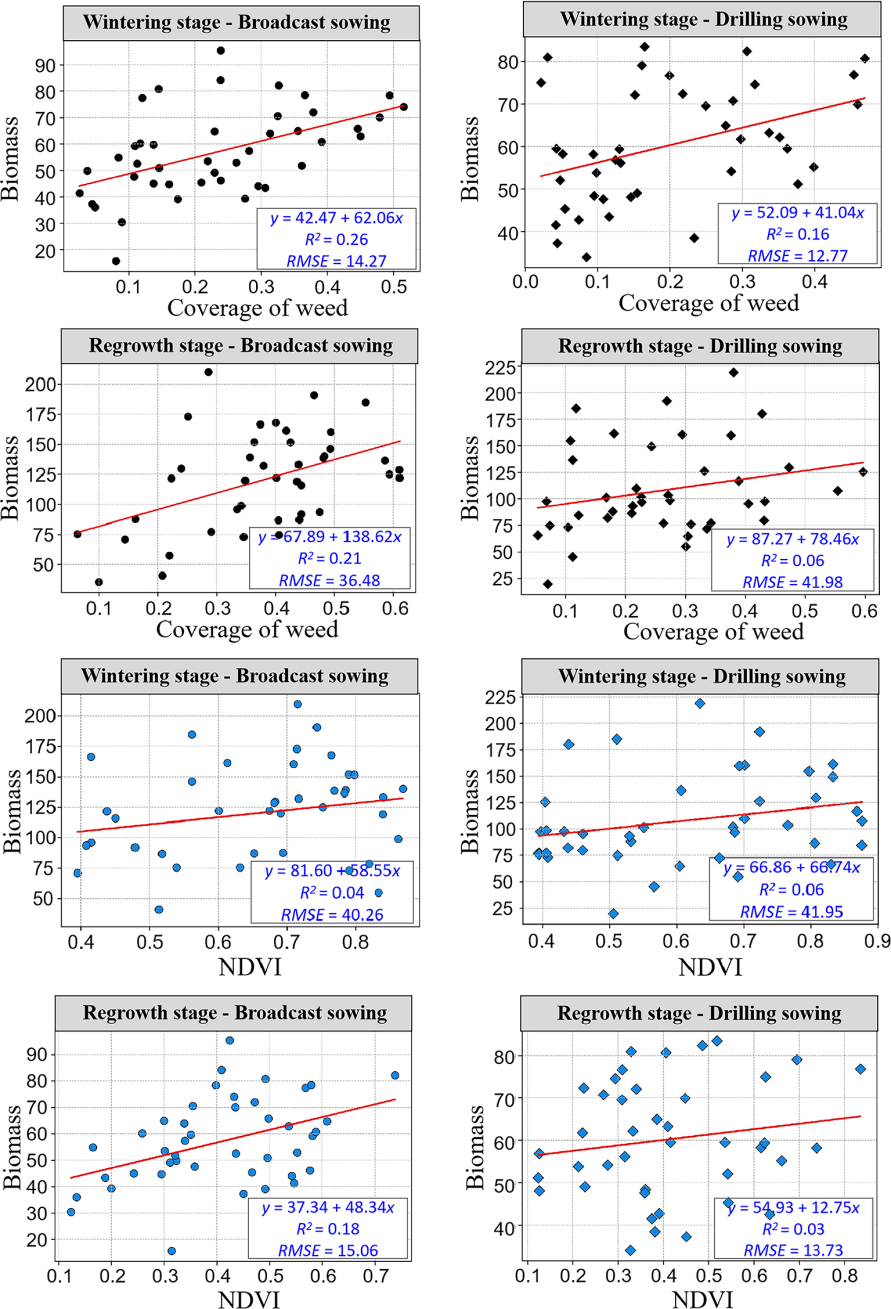
The estimation of weed biomass using canopy cover or vegetation indices under different conditions. The unit of the RMSE is kg·ha^-1^

We analyzed the spectral curves and common characteristic parameters of wheat and weeds. As shown in Fig. 5, the curves of weeds and wheat in the 400–1000 nm band are very similar, and there are some differences only in the green and red regions of visible light. Furthermore, there is a large amount of overlap in other areas, making it challenging to distinguish weeds and wheat by spectrum. In accordance with previous research advancements, vegetation cover and canopy vegetation indices such as NDVI are closely related to vegetation biomass. Biomass regression models constructed using cover and NDVI typically exhibit a high level of accuracy. However, as evidenced by the results of this study (Fig. 6), it can be observed that the biomass regression models developed using canopy cover and NDVI for weed estimation yield relatively poor performance. Notably, the estimation accuracy is relatively better during the overwintering period, although the RMSE values exceed 10, and they escalate to over 40 during the regreening period. Overall, the estimation performance using canopy cover is slightly superior to that of NDVI. Several factors contribute to the suboptimal estimation performance, including: (1) The DeepLab model used for weed cover estimation cannot distinguish the extent of weed infestation, and it can only differentiate between the presence and absence of weeds; (2) When calculating weed biomass using vegetation indices, it is challenging to differentiate between weeds and wheat. The calculation of biomass is influenced by wheat, especially during the regreening period, where increased biomass leads to larger errors.

### Semantic segmentation effect of weeds

There was still some overlap between wheat and weeds in the orthophotos during the wintering stage, which resulted in large errors when attempting to distinguish weeds directly from wheat. In this research, five semantic segmentation methods were used to distinguish weeds, and the results were shown in Table 2. Most models have high recognition accuracy for cultivated land, with IoU values above 90; the UNet model was found to have the highest IoU value. However, models other than DeepLabV3 + have poor recognition effects on weeds and wheat. It can recognize weeds and wheat with an MIoU value of over 85, which can identify weeds and wheat more accurately. The ResNet-38 model has the shortest processing time per image, while the PSPNet has the longest. The processing time for DeepLabV3 + is 46.5ms, which is within an acceptable range.

**Table 2:** The detection results of different models

Method	Weed	Wheat	Land	MIoU	Recall	F1	Time (ms)
ResNet-38_MS_COCO	67.2	71.6	91.3	76.7	89.3	76.7	30.6
PSPNet	61.5	68.6	92.5	74.2	90.7	73.3	76.7
DeepLabV3	70.6	70.3	94.2	78.4	90.5	79.3	37.6
UNet	77.4	79.5	95.8	84.3	94.2	85.1	39.2
DeepLabV3+	83.3	87.5	95.6	88.8	90.6	96.8	46.5

**Fig. 7:**
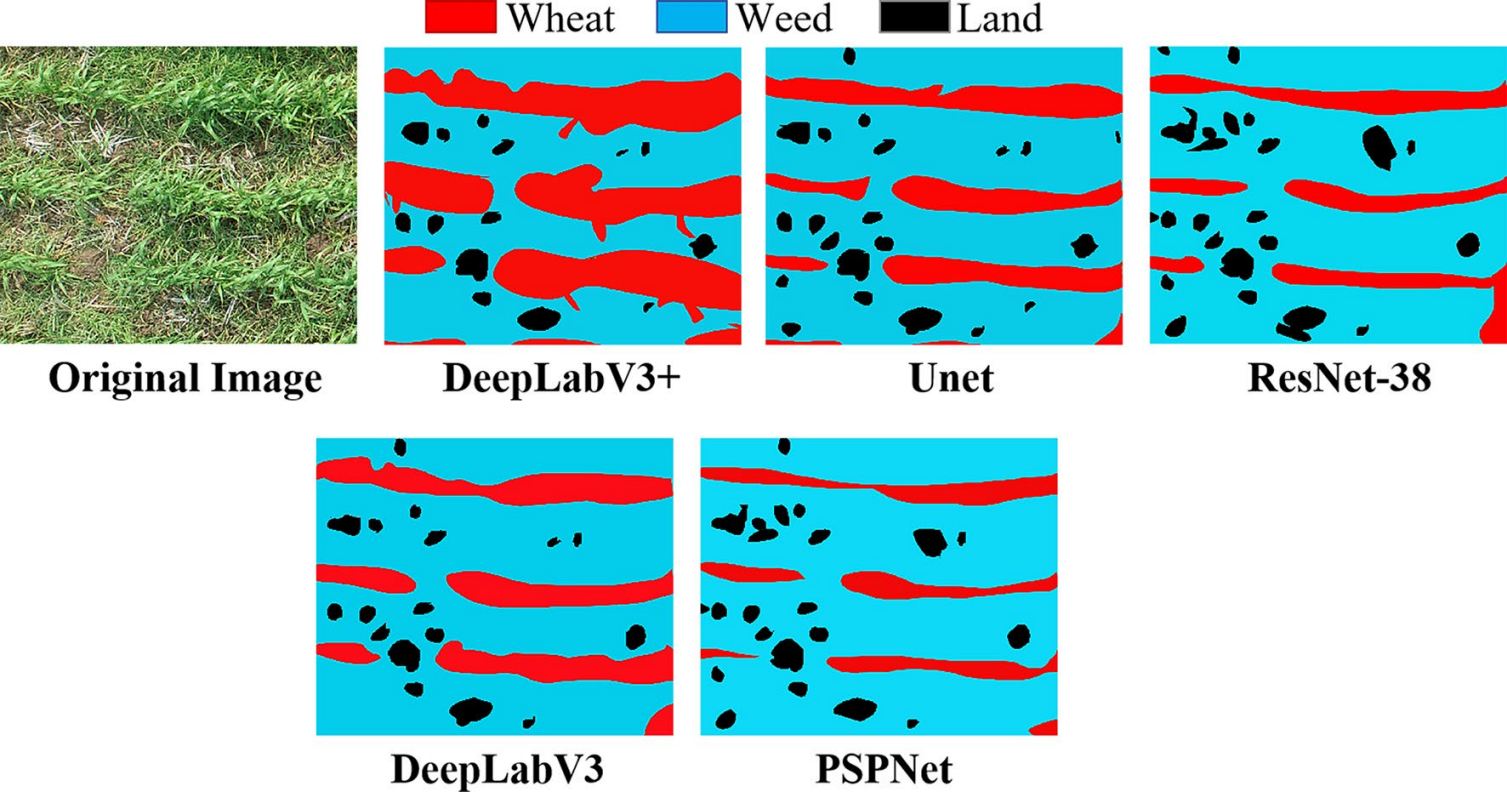
The semantic segmentation performance of different models

As shown in Fig. 7, the segmentation performance of different models on the original RGB images indicates that DeepLabV3 + achieves the best results, accurately identifying almost all wheat and weeds, with good performance in the edge regions of the wheat. The main issue with the lower accuracy of the other models is the high rate of missed wheat identification and the high rate of weed misidentification. PSPNet, which performs the worst, can only identify the central regions of row-planted wheat, with the edge areas almost entirely misidentified as weeds. Additionally, the models show little difference in their ability to identify land, with all models accurately recognizing land.

**Fig. 8:**
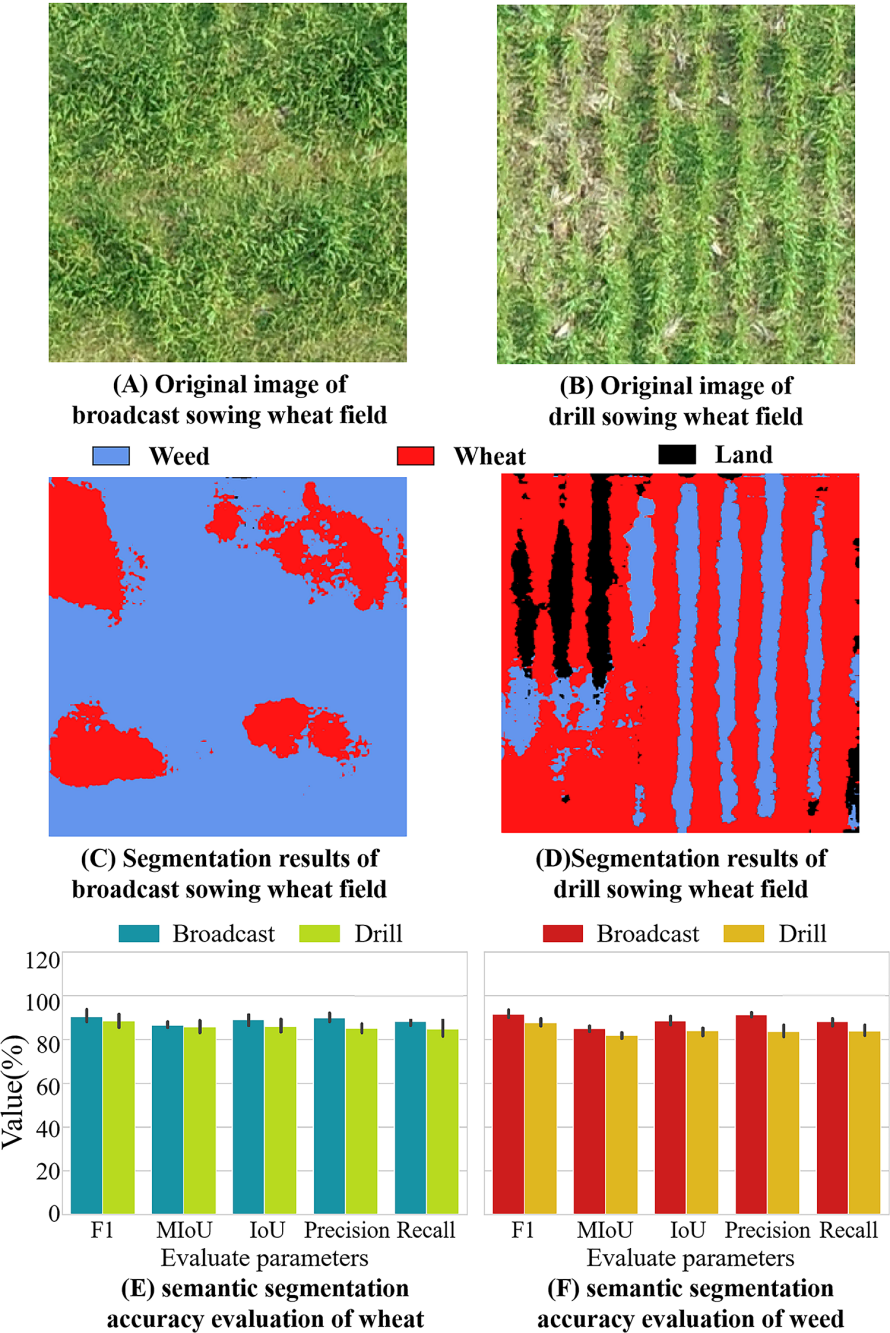
Segmentation results of DeepLabV3+. (**A**) and (**B**) are the original images of the broadcast sowing wheat field and drilled sowing wheat field; (**C**) and (**D**) are the segmentation results; (**E**) and (**F**) are the segmentation accuracy values of wheat and weeds

The choice of seeding method and different growth stages have an impact on the results of the semantic segmentation of weeds (Fig. 8). The results showed that the F1, MIoU, precision, and Recall values of the model for weed and wheat monitoring were higher than 85%. The segmentation accuracy of the model for wheat was slighter than that of weeds. What’s more, we found that sowing methods had a greater influence on the segmentation results than under different growth stages, with scattered wheat fields showing higher precision in detecting both wheat and weeds. The segmentation precision value for the scattered wheat was 91.27%, whereas for drilled wheat, it was only 87.51%. In weeds, both missing and improper segmentation occurred in the edge areas regardless of the sowing method.

**Fig. 9:**
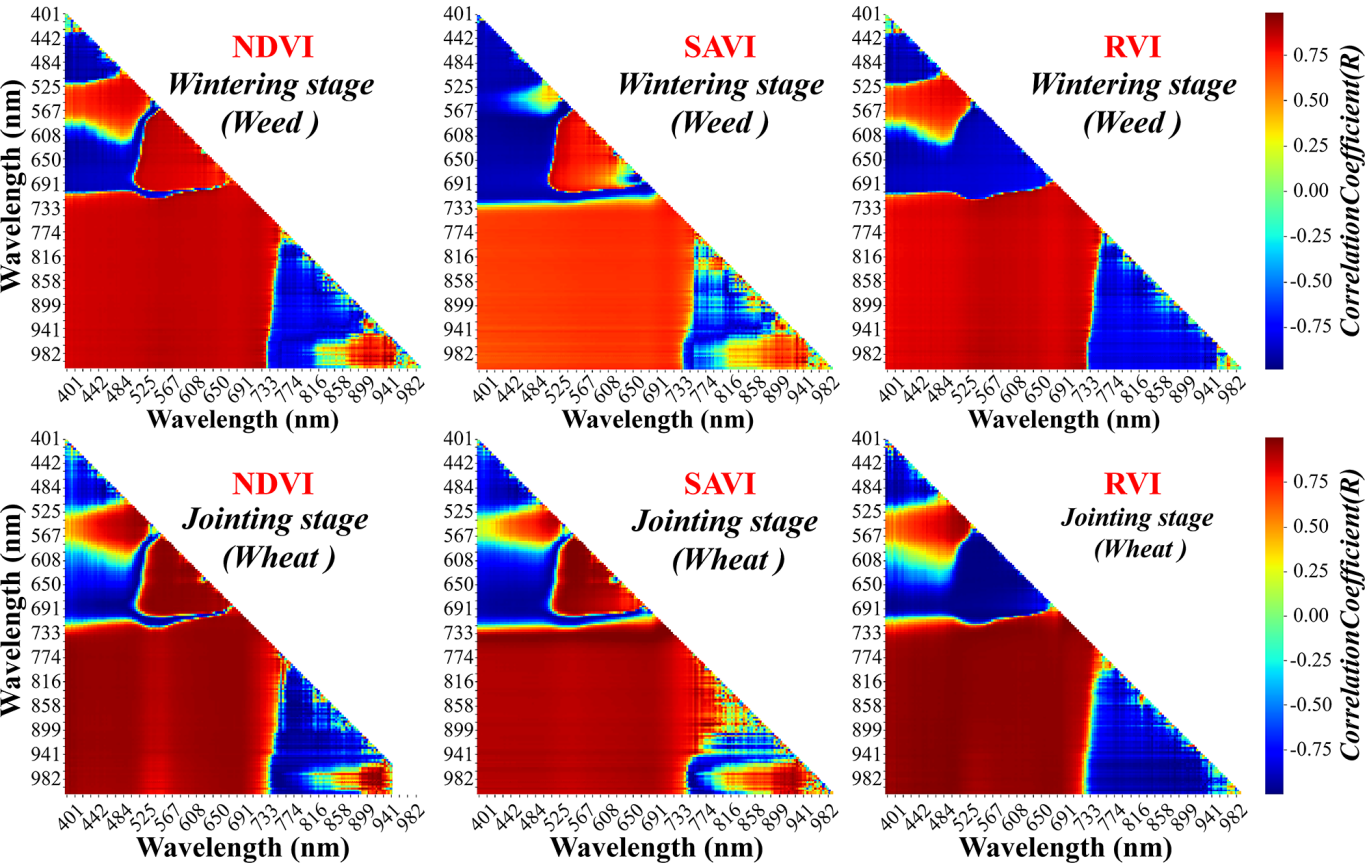
Correlation analysis between spectrum and biomass of weeds and wheat

The DeepLabv3 + model exhibits clear advantages in distinguishing weeds from wheat, but it is unable to determine the amount of weeds and formulate preventive measures. The vegetation index has been proven to be highly reliable in estimating biomass. As shown in Fig. 9, the common vegetation index correlates well with wheat and weeds biomass in different growth periods. Notably, the NDVI shows a higher correlation than other vegetation indexes. The (960 nm, 733 nm) combination of NDVI combination has the highest correlation with wheat during the wintering period, whereas the (960 nm, 719 nm) combination has the highest correlation with weeds during the same period. Similarly, the (960 nm, 710 nm) combination of NDVI has the highest correlation with wheat during the jointing period, while the (960 nm, 700 nm) combination has the highest correlation for weeds during the same period. The correlation between the combinations mentioned above and their respective biomass all exceeds 0.92, indicating that they can be utilized to estimate the corresponding biomass.

### Estimation of weed biomass

**Fig. 10:**
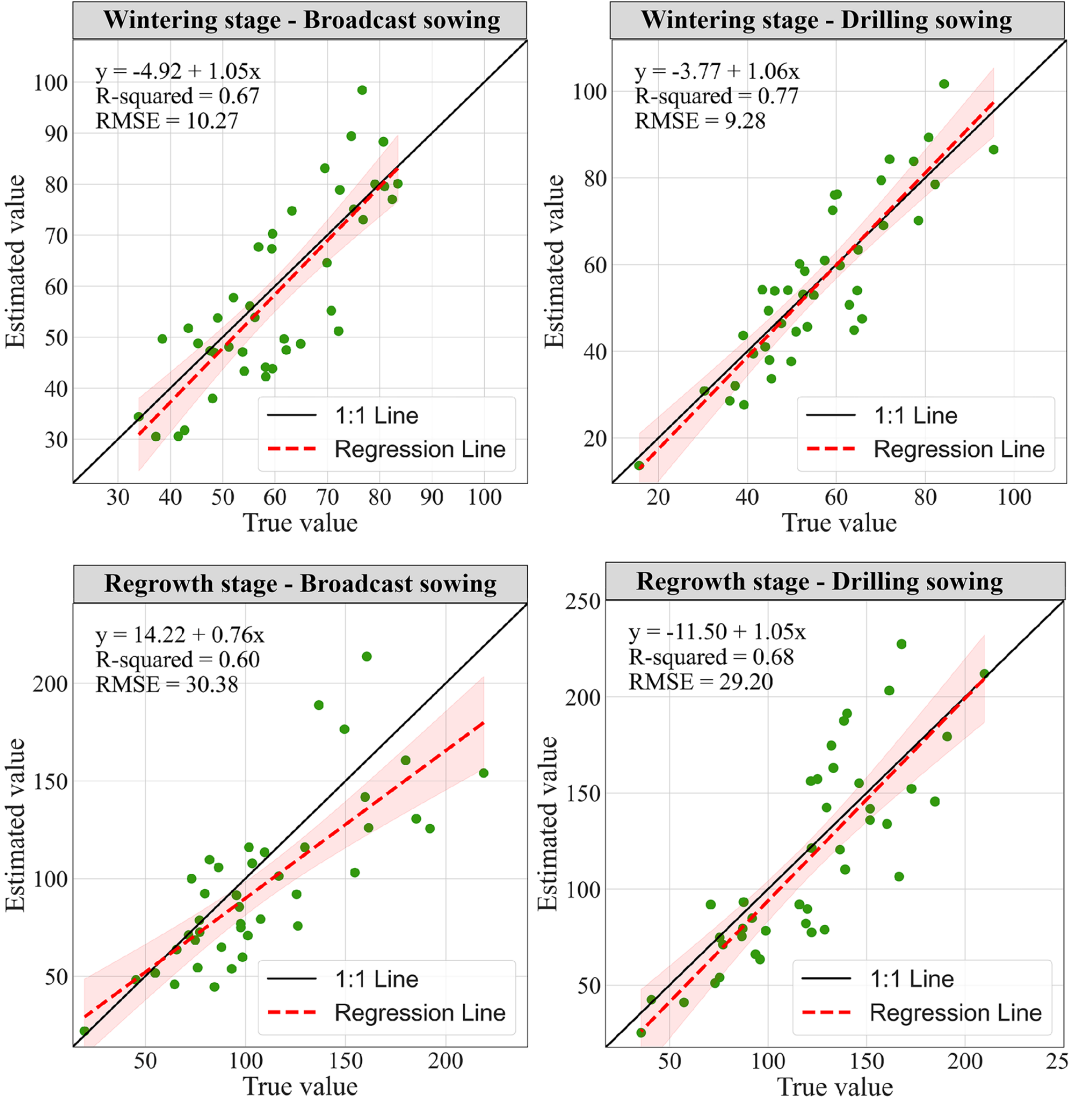
Model testing results using an independent dataset. The unit of RMSE is kg·ha^-1^

Considering the difficulty of solely utilizing CC and vegetation indices to estimate weed occurrences, and recognizing the strong correlations between CC and multiple vegetation indices with weed biomass, this study employed CC and several vegetation indices as input variables for SVR models to assess weed occurrences during the wintering and regrowth stages of both drilled and broadcast wheat. Remarkably, favorable results were obtained, with modeling R^2^ consistently exceeding 0.85. Weed biomass estimation during the wintering period demonstrated higher precision, with modeling RMSE remaining below 8 kg·ha^-1^ for both drilling and broadcasting scenarios. However, during the regrowth stage, due to the increased wheat population, modeling precision slightly diminished, with RMSE values not exceeding 20 kg·ha^-1^. Model validation was conducted on independent samples, as depicted in Fig. 10. The precision of weed biomass estimation substantially improved for both seeding methods and growth stages. During the wintering period, weed biomass estimation exhibited superior accuracy compared to the regrowth stage, with an average RMSE of only 40.03 kg·ha^-1^. Among the winter scenarios, weed estimation accuracy was highest for drilled conditions, with an RMSE of only 9.28 kg·ha^-1^. Conversely, under broadcast conditions during the regrowth stage, weed biomass estimation was the least accurate, with an R^2^ of only 0.6 and an elevated RMSE of 30.38 kg·ha^-1^. In summary, weed monitoring accuracy was generally higher under drilled conditions, with an average RMSE 2.17 kg·ha^-1^ lower than that under broadcast conditions.

### The impact of weeds

**Fig. 11:**
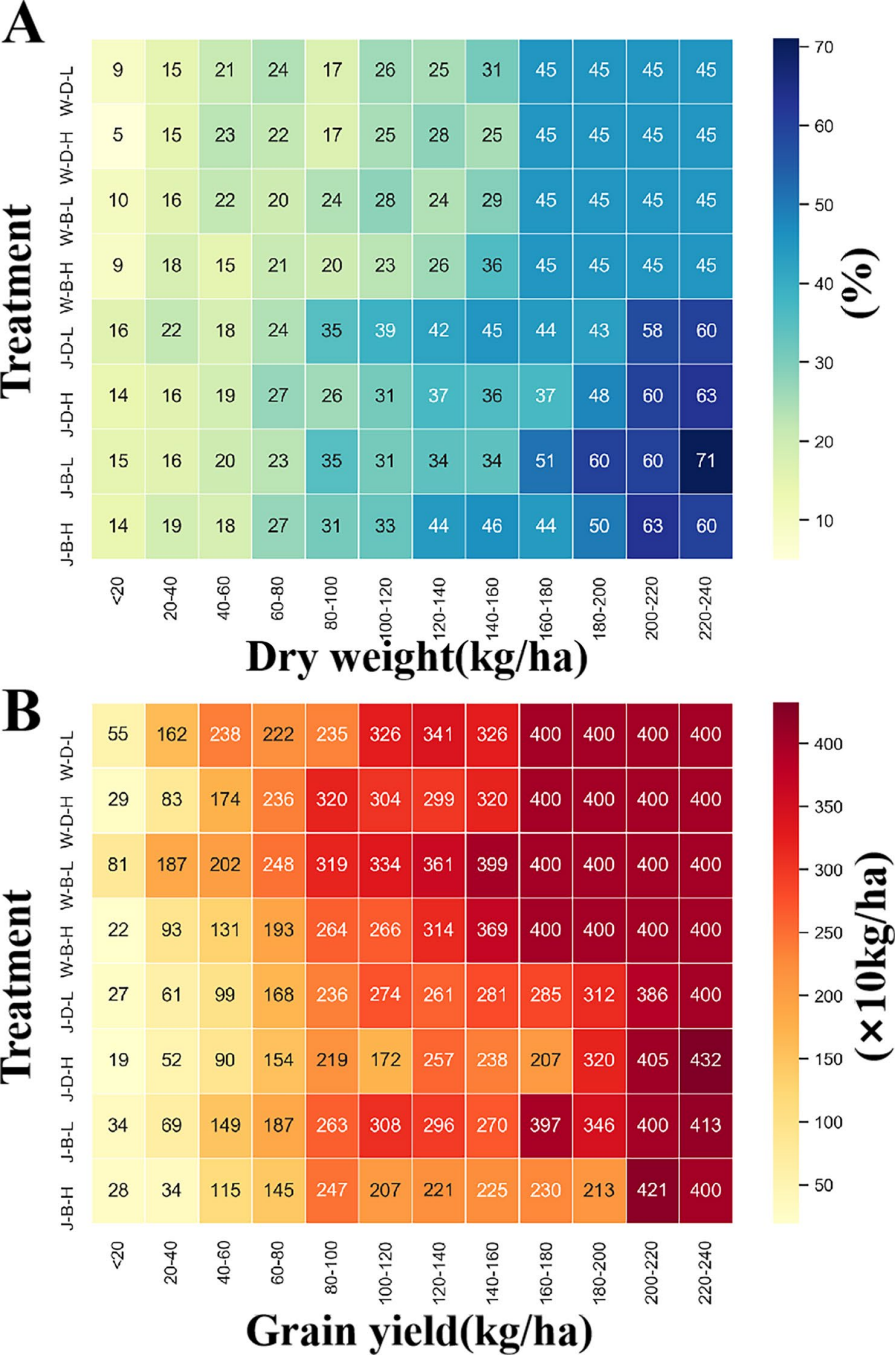
Effects of weeds on wheat growth. (**A**) The effects of weeds on wheat dry weight, (**B**) The effects of weeds on wheat grain yield. J: jointing stage, W: wintering stage, B: Broadcast sowing, D: drill sowing, L: plant density of 180 × 104 plant·ha^-1^, H: plant density of 300 × 104 plant·ha^-1^

To further clarify the effects of weeds on wheat, we analyzed the impacts on wheat dry weight and grain yield under different treatments. As shown in Fig. 11, an increase in the number of weeds led to a corresponding decrease in wheat dry weight, with a maximum reduction of 71% compared to areas without weeds. Similarly, the number of weeds also harmed yield, with a maximum reduction of 4320 kg·ha^-1^, or a 60% drop. However, the impact of weeds varied depending on the sowing methods. Planting density and growth stages. Specifically, scattered wheat was more susceptible to weeds than drilled wheat, and wheat in high density was more affected than in low density. Additionally, weeds that emerged during the wintering stage had a greater impact on wheat yield.

## Discussion

The identification of farmland weeds has always been a research focus in smart agriculture. Traditional methods primarily rely on extracting image color, morphology, and texture features [5, 18], or distinguishing weeds and crops through morphological features [19]. However, wheat field weeds, especially grass weeds and wheat, share similar features, making it difficult to differentiate them. Gašparović et al. used UAV images to distinguish between oats and weeds [20]. They found that weed identification methods based on color features have large limitations, especially when the crop and weeds are similar in color. Moreover, due to the color and spectral similarities between wheat and weeds, it is not easy to successfully distinguish the two using conventional machine learning classification methods. Consequently, scholars have turned to deep learning algorithms to identify farmland weeds. For instance, the SegNet segmentation network has been used to distinguish sugar beets and weeds [21], convolutional neural networks have been used to distinguish crops and weeds during the seedling period [22], and deep convolutional neural networks have been used to identify weeds among rapeseed rows [23]. However, these methods are limited to the seedling stage, where the difference between weeds and crops is more significant, and the images are mostly collected near the ground. Similar characteristics of weeds and wheat make monitoring weeds with UAVs more difficult. Considering that the convolutional neural network model can better identify weeds [24], the filtered convolution structure can identify weeds in UAV images.

Therefore, this study constructed a comprehensive wheat field weed dataset by acquiring images from multiple locations. What’s more, given the complexity of precisely distinguishing these regions, some boundary inaccuracies between these classes were unavoidable when preparing the dataset. The primary focus was on maximizing weed identification while minimizing the labeling of wheat and bare soil. This strategy was chosen to reduce the risk of missed weed detection, even if it led to a higher false positive rate. In practical weed management, intervention is usually required only when weed populations reach a certain threshold, making false positives less critical than missed detections. We selected several classical semantic segmentation models based on this dataset to perform weed segmentation in wheat fields. To ensure the accuracy of the segmentation results, this study compared the performance of different models and continuously tuned parameters, ultimately optimizing the DeepLabV3 + model, which exhibited the best performance for weed segmentation in wheat fields. This model utilizes an encoder-decoder architecture, where the encoder architecture uses DeepLabV3, and the decoder uses a simple but effective module to restore the target boundary details. It can use expanded convolution to control the resolution of features under specified computing resources, resulting in better weed identification accuracy. The model used in this research can identify weeds in wheat fields and has an MIoU value of 88.8.

The findings of this study demonstrate that, with proper tuning and adequate training, classical semantic segmentation models can effectively segment wheat field weeds and achieve satisfactory accuracy. However, these classical models have certain limitations. For instance, they typically require large datasets for effective training, and their model sizes are relatively large, which may pose challenges in practical applications. Future research could build upon the results of this study by further optimizing these classical models to reduce the training data requirements and decrease the model size, thereby enhancing their efficiency and applicability in real-world scenarios.

The convolutional neural network has clear advantages in distinguishing field weeds and wheat, but it is not effective in evaluating the amount and degree of damage of weeds. To realize the precise prevention and control of weeds, improve the UAV plant protection spray efficiency, and protect the ecological environment, it is necessary to construct a distribution map of the occurrence of weeds in the field. Although previous studies have proposed methods for estimating vegetation biomass with RGB images [25, 26], the estimation of weed biomass in wheat fields based on RGB images is not satisfactory due to the sowing methods and the interaction between weeds and wheat. After analysis, some spectral indexes have very significant correlations with the biomass of wheat and weeds. After locating the weed occurrence area, we extract the spectral characteristics of weed areas, use the vegetation index to estimate the occurrence of weeds, and evaluate its image of wheat growth. If only hyperspectral UAVs are used to obtain images, and then weeds are classified, the flying height of the UAV must be set below 25 m to effectively train the DeepLabV3 + model, which will result in low image acquisition efficiency. Therefore, our research combined the images taken by hyperspectral UAV and the small RGB UAV to achieve the highest image acquisition efficiency. This study utilized a combination of hyperspectral cameras and high-definition cameras to monitor the biomass of graminaceous weeds in wheat fields. The reasons include: graminaceous weeds in wheat fields have a high similarity to wheat in terms of spectral and color characteristics, but they can be distinguished based on leaf structure and other texture information. These details can only be captured by high-definition cameras. If deep learning algorithms are to be used for the identification of graminaceous weeds in wheat fields, it is necessary to obtain canopy images with high-definition cameras. While hyperspectral cameras can effectively reflect the biomass of crop populations, most current hyperspectral cameras do not have a high enough resolution, making it difficult to distinguish between graminaceous weeds and wheat through spectral images. This research has confirmed that the 960 nm and 710 nm bands are closely related to the biomass of graminaceous weeds. In the future, with the improvement of the resolution of spectral sensors, it will be possible to estimate the occurrence of graminaceous weeds using only multispectral drones.

The method proposed in our study can realize the monitoring of the occurrence of weeds in wheat fields, but there is still no better way to solve the occlusion problem. According to experimental surveys, different wheat planting densities, sowing methods, and the occlusion of wheat on weeds during growth periods are different. In the wintering period, the occlusion of wheat on weeds generally did not exceed 5%, while the occlusion increased in the jointing period but generally did not exceed 10%. Increasing the planting density from 180 × 104 plant·ha^-1^ to 300 × 104 plant·ha^-1^ resulted in a 3% increase in occlusion during the wintering stage, with a maximum occlusion of 20% during the jointing stage. The wider row spacing of drilled wheat led to heavier weed occlusion compared to scattered wheat. The occlusion issue affects the accuracy of weed monitoring, but it has less impact on the occurrence of weeds and fixed-point weeding. Therefore, we did not collect further data pertinent to the occlusion issue.

## Conclusions

This study developed a method for monitoring weed occurrence in wheat fields using deep learning algorithms and spectral analysis techniques. The findings indicate that traditional image attributes such as color and texture pose challenges in distinguishing grass weeds in wheat fields. However, the application of deep learning algorithms significantly enhances weed identification accuracy. Among the evaluated algorithms, DeepLabV3 + demonstrated superior performance, surpassing segmentation methods like UNet and PSPNet. By selecting sensitive NDVI bands, weed biomass can be effectively estimated, establishing a robust framework for assessing the impact of weed infestations. The deep learning-based weed canopy cover identification model in this study accurately estimates weed canopy cover. Furthermore, integrating canopy cover with vegetation indices enhances the precision of weed biomass estimation. This proposed method facilitates efficient UAV-based weed monitoring in wheat fields and the development of targeted plant protection strategies, providing technical support for UAV and precision plant protection.

## Data Availability

Data will be made available on request.
